# Curcumin and Gut Microbiota: A Narrative Overview with Focus on Glycemic Control

**DOI:** 10.3390/ijms25147710

**Published:** 2024-07-14

**Authors:** Simona Servida, Alessandra Piontini, Francesca Gori, Laura Tomaino, Gianluca Moroncini, Vito De Gennaro Colonna, Carlo La Vecchia, Luisella Vigna

**Affiliations:** 1 Obesity and Work Centre, Occupational Medicine Unit, Clinica del Lavoro L. Devoto, Fondazione IRCCS Ca’ Granda Ospedale Maggiore Policlinico, 20122 Milan, Italy; simona.servida@alice.it (S.S.); alessandra.piontini@policlinico.mi.it (A.P.); vito.degennaro@policlinico.mi.it (V.D.G.C.); 2 Department of Anesthesia, Critical Care and Emergency, Fondazione IRCCS Ca’ Granda Ospedale Maggiore Policlinico, 20122 Milan, Italy; francesca.gori@policlinico.mi.it; 3 Postgraduate School of Emergency Medicine, Università Politecnica delle Marche, 60121 Ancona, Italy; l.tomaino@pm.univpm.it; 4 Department of Clinical and Molecular Sciences, Università Politecnica delle Marche, 60121 Ancona, Italy; g.moroncini@univpm.it; 5 Department of Clinical Science and Community Health, DISSCO, Università degli Studi, 20122 Milan, Italy; carlo.lavecchia@unimi.it

**Keywords:** curcumin, hypoglycemic effect, gut microbiota

## Abstract

Turmeric is a spice widely used in China, Southeast Asia, and in traditional Ayurvedic medicine. Its safety profile and efficacy as an antioxidant, anti-inflammatory, antimicrobial, antitumor, antidiabetic, and anti-obesity agent have led to extensive research into its potential role in preventing and treating metabolic diseases. The active compound in turmeric is curcumin, which exhibits low systemic bioavailability after oral administration. However, it is detectable in the gut, where it bidirectionally interacts with the gut microbiota (GM), which plays a crucial role in maintaining host health. The favorable effects of curcumin, particularly its hypoglycemic properties, are linked to alteration in intestinal dysbiosis observed in type 2 diabetes mellitus and metabolic syndrome patients. Restoration of the eubiotic GM may contribute to glycemic homeostasis. Preclinical and clinical studies have demonstrated the involvement of the GM in the regulation of glucose and lipid metabolism. Although the underlying mechanism remains incompletely understood, intestinal dysbiosis is associated with insulin resistance, hyperglycemia, and low-grade inflammation. In the present overview, we summarize the biological properties of curcumin, focusing on its link with GM and, therefore, on its potential role in metabolic diseases.

## 1. Introduction

*Curcuma longa* is a perennial, rhizomatous herbaceous plant belonging to the *Zingerbaceae* family. Its root consists of a large yellow-orange, aromatic rhizome, which, when dried and ground, becomes a powder, commonly called turmeric, widely used as a spice. It has been used for centuries in China, Southeast Asia, and in traditional Ayurvedic medicine. Due to its safety efficacy as an antioxidant, anti-inflammatory, antimicrobial, anti-tumor, anti-diabetic, and anti-obesogenic agent, it is studied with potential use in the prevention and treatment of metabolic diseases. Its active component, curcumin, is poorly bioavailable at a systemic level after oral administration, while it is found in the intestine where it meets the gut microbiota (GM), with which it establishes a bidirectional relationship. Curcumin is transformed at the enteric level by the GM into pharmacologically more active metabolites. In turn, its metabolites facilitate and influence the growth of favorable strains of the GM. The GM is closely linked to the health of the host. Thus, the therapeutic effects of curcumin, and among these the hypoglycemic one, are linked to the changes in the dysbiosis that characterized type 2 diabetes mellitus (T2DM) and the metabolic syndrome (MS), and thus to the restoration of a eubiotic microbiota, capable of balancing the glucose homeostasis. Although several clinical studies demonstrate how GM is widely involved in the regulation of glucose and lipid metabolism and how dysbiosis causes insulin resistance, hyperglycemia, and chronic low-grade inflammation, the mechanism is still poorly understood. This qualitative narrative review provides an overview of the bidirectional interaction between turmeric and GM, highlighting the influence that metabolites derived from curcumin itself, formed following its metabolization and fermentation by GM, have on the host glycemic homeostasis. The search was conducted in PubMed in 2024. The keywords of the search were “curcumin”, “gut microbiota”, and hypoglycemic effect, and in vitro, preclinical, and clinical studies and reviews reporting results on the interaction of curcumin/gut microbiota were included.

## 2. *Curcuma longa*

Turmeric rhizome powder, mainly present in mixtures of aromatic spices such as curry, is a polyphenol characterized by an aromatic ring structure with at least a hydroxyl group, which derives from the shikimic acid pathway [1,2]. The most representative polyphenolic constituent of turmeric is curcumin [1,7-bis(4-hydroxy-3-methoxyphenyl)-1,6-heptadiene-3,5-dione], also known as “Curcumin I”. The molecule is symmetrical, with two similar aromatic rings and conjugated double bonds that make it a very effective electron donor, essential against superoxide radicals. However, there are two other compounds known as curcumin, namely “Curcumin II” (demethoxycurcumin) and “Curcumin III” (bisdemethoxycurcumin), which differ in the number of methoxy groups on the aromatic ring. They represent 10–20% and 3% of the total curcuminoids, respectively, with different pharmacological activities [1,2]. In this overview, the focus will be on Curcumin I, hereafter simply referred to as curcumin, as it is the most abundant compound of the three forms. Curcumin is almost completely insoluble in water but is readily soluble in organic solvents, including acetone and ethanol [2,3].

The commercial form is available as a standardized curcuminoid preparation containing 80% curcumin, 15% methoxycurcumin, and 5% bisdemethoxycurcumin [3,4].

**Figure 1:**
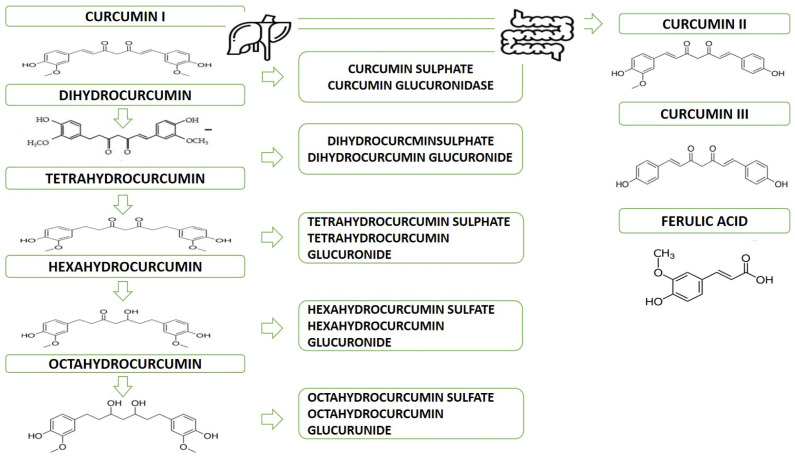
Curcumin metabolism after oral intake. The metabolites of curcumin derived from the reduction (phase I) and conjugation reactions (phase II) are represented. Sulfatation and glucuronidation are the main pathways between conjugation reactions.

In humans, after oral administration, only glucuronide and sulfate derivatives can be found in the blood, while whole curcumin is present only in low and limited doses [2]. Its low availability, mainly due to poor absorption, limited tissue distribution, and rapid metabolism, means that the therapeutic efficacy of curcumin is still debated [1]. Curcumin orally ingested passes unchanged through the stomach (stable in a pH range of 2.5 to 6.5) and only in the large intestine undergoes phase I and phase II metabolizations [1,2,3]. In phase I, the reductases introduce a series of polar and reactive groups into the substrate. The reduction of four double bonds of heptadiene-3,5-dione results in the reduction of curcumin to dihydrocurcumin (DHC), then to tetrahydrocurcumin (THC) (reduced curcumin analog and found mainly in the gastrointestinal tract), and finally to hexahyrocurcumin and octahyrocurcumin. This type of transformation occurs mainly in enterocytes and hepatocytes [1,2,3,5,6]. The metabolites formed in phase I are transported to the intestinal and hepatic cytosol, where they are transformed (phase II) into conjugate derivatives (i.e., conjugated curcumin, conjugated DHC, conjugated THC, hexahydrocurcumin, and conjugated octahydrocurcumin). Figure 1 shows the metabolism of curcumin in phases I and II.

**Figure 2:**
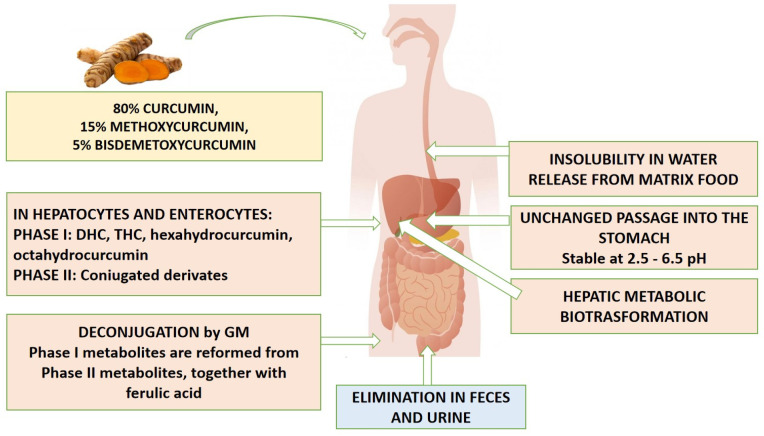
Curcumin pharmacokinetics after oral intake. DHC—Dihydrocurcumin; THC—Tetrahydrocurcumin.

The main and predominant route of conjugation is represented by glucuronidation. Curcumin glucuronides represent the main metabolites present in body fluids (about 99% of the plasma curcumin) in organs and cells despite being less active molecules than their progenitors and with a greater molecular weight [2,6]. Then, the GM is able to deconjugate phase II metabolites and reconvert them into the corresponding phase I metabolites, also producing ferulic acid [6]. Curcumin and its derivatives are therefore mainly excreted through feces [1]. Figure 2 shows the curcumin kinetic after oral intake.

After oral administration, curcumin peak blood concentrations are observed within 1 to 2 h and become undetectable after approximately 12 h [1]. A high dose administrated in humans, equal to 8 g/kg/day, did not allow detectable levels [7]. A clinical study that saw the oral administration of 3.6 g curcumin revealed a plasma peak of 11.1 nmL/L 1 h after administration [2,3].

The poor curcumin availability after oral administration would limit its pharmacological potential and, consequently, its clinical application. Therefore, new formulations have been designed to improve bioavailability. These delivery systems included β-micelles, nanoparticles, liposomes, nanoemulsions, phospholipid complexes, and nanobubbles [1,2,8,9]. Co-administration of natural UDP-glucuronyl transferase inhibitors, such as silybin, quercetin, and piperine, may also increase the curcumin bioavailability [1,10]. The food matrix also influences the adsorption of polyphenols. Bioavailability appears to be superior when consumed as fresh or dried powder compared to turmeric found in multi-compound supplements [1,2]. Other factors that influence bioavailability are the processing of the rhizome, which includes heating, drying, and grinding phases and together with them, the culture conditions, such as climate, stress suffered by the plants, irrigation, etc. [6] Further, women adsorb curcumin 1.4 times more than men, probably due to a greater expression and activity of some liver enzymes [6,10,11].

## 3. Effects of Gut Microbiota on Curcumin

Although the bioavailability in the blood is limited after oral administration, high concentrations of curcumin have been found in the gastrointestinal tract [2]. This is close contact with GM, and curcumin seems to have established a bidirectional relationship. In fact, curcumin is biotransformed by GM into active metabolites and, at the same time, carries out a regulatory activity on the GM itself. This close relationship could explain the inconsistencies between the poor bioavailability of the native active ingredient and the pharmacological effect of curcumin observed in the organism. The curcumin metabolic transformation does not appear to occur only by the enterocyte and hepatocyte enzymes but also by the microbial origin enzymes coming from the GM. The biological activity of gut-derived metabolites differs from that of native curcumin, and their properties are specific and often more potent [1,2]. Therefore, the GM composition influences the bioavailability of curcumin metabolites. Furthermore, the response to curcumin administration is individual: the oral curcumin intake depends on the ability of the GM belonging to the individual to metabolize the polyphenol. In the reviews by Ng et al. [12], which analyzes all the articles published between 1998 and 2020, and by Scazzocchio et al. [2], which includes the studies published on PubMed in five years, from 2015 to 2020, the regulatory effect of the GM on curcumin is also confirmed thanks to the presence of its metabolites found in the analyzed feces [2,12]. 

### In Vitro, Preclinical, and Clinical Studies

Table 1 gives a summary of the studies regarding the action of GM on curcumin.

Lou et al. [13] evaluated in vitro the transformation of curcumin (90% purity, 100 µM) added to a culture of intestinal bacteria from human fecal samples, obtaining 23 different metabolites from the polyphenol. The new compounds come from bacterial fermentation through different metabolic pathways, such as acetylation, hydroxylation, demethylation, or a combination of these [2,13]. Burapan et al. [14] added a mixture of curcuminoids (10 nM) consisting of curcumin, demethoxycurcumin, and bisdemethoxycurcumin to fecal samples of three healthy volunteers in mixed cell cultures. In the samples in which the *Blautia* spp. strain (MRG-PMF1) was present, and two metabolites were obtained by demethylation, namely bis-demethylcurcumin and demethylcurcumin [2,14]. Again in vitro, Tan et al. [15] noted that in contact with human fecal starters, after 24 h, 24% of the curcumin, 61% of the demethoxycurcumin, 97% of the bisdemethoxycurcumin were degraded in three main metabolites: THC, dihydroferulic acid, and 1-(4-hydroxy-3-mehtoxiphenyl)-2-propanol [2,15]. Sun et al. [16] administered curcumin to transgenic mice with Alzheimer’s disease by gastric gavage and found it in a modified form. In fact, the GM, through reduction, demethylation, demethoxylation, and hydroxylation reactions, had created eight metabolites with neuroprotective capabilities. These included hexahydrocurcumin, demethoxylated curcumin, DHC, hexahydrocurcumin, and demethylated hexahydrocurcumin [2,6,16]. Peron et al.’s study [17] on healthy volunteers evaluated the urinary composition after 28 days of 100 mg of curcuminoids administration, detecting the presence of curcumin and two metabolic derivates, THC and DHC. The appearance of urinary metabolites confirmed the adsorption of the curcuminoid and its metabolism at both liver and enteric levels [6,12,17].

Once the presence of the urinary and fecal curcumin metabolites has been established, it is necessary to identify which species of GM are capable of transforming the polyphenol into the corresponding derivatives. According to Hassaninasab et al. [18], the microorganism with the highest capacity to metabolize curcumin is *E. coli*, which is able to obtain THC from native curcumin. The enzyme used by *E. coli* to metabolize curcumin into THC was identified and named NADPH-dependent curcumin/dihydrocurcumin reductase [2,6,18,19]. In turn, the firmicute *Blautia* spp., demethylating curcumin, gives rise to demethylcurcumin and bisdemehylcurcumin. *B. longum BB536*, *B. pseud catenulaum G4*, *E. faecali JCM 5803s*, *E. coli K-12*, *L. acidophilus* and *L. casei* metabolize curcumin obtaining a metabolism equal to 50% of the original compound [2,3,6,20]. Similarly, *E. fergusonii ATCC 35469*, *E. coli ATCC 8739*, and *E. coli DH10B* strains produce DHC, THC, and ferulic acid [3,15]. The bacterial strain of *B. megaterium DCMB-002* also creates new metabolites through metabolic pathways that include hydroxylation, demethylation, reduction, and demethoxylation [3,20,21]. *P. anomal*, instead gives rise to 4 metabolites, including 1,7-bis(4-hydroxy-3-methoxyphenyl) hepta-3,5 diolo, 5-hydroxyi-1,7-bis (4-hydroxy-3 methoxyphenyl) hepata-3-one, 5-hydroxy-1,7-bis (4-hydroxyphenyl) hepata-3-one e 5-hydroxy-7-(4-hydroxy-3-methoxyphenyl)-1-(4-hydroxyphenyl) hepta-3-one [20,22,23,24].

**Table 1:** In vitro, preclinical, and clinical studies of the effects of the gut microbiota on curcumin.

References	Curcumin Dose and Model	Gut Microbiota and Reactions	Metabolites
Lou et al. [13]	Curcumin >90% purity Addition to human fecal bacteria	Human fecal bacteria Acetylation, hydroxylation, demethylation, combination of these	23 different metabolites (>hexahydroxycurcu-min glucuronide)
Burapan et al. [14]	Mixture of curcuminoids (curcumin, demethoxycurcumin, bisdemethoxycurcumin) Addition to human fecal bacteria in mixed cell cultures	*Blautia* sp. (*MRG-PMF1*) Demetilation	Demethylcurcumin, bisdemethylcurcumin
Tan et al. [15]	Mixture of curcuminoids (80.1% curcumin, 15.6% demethoxycurcumin, 2.6% bisdemethoxycurcumin) in vivo incubation	*Escherichia fergusonii ATCC 35496, Escherichia coli ATCC 8739, Escherichia coli DH10B* Reduction by NADPH-depend curcumin/dihydroxycurcumi reductase enzyme	Tetrahydrocurcumin, dihydroferulic acid, 1-(4-hydroxy-3-methoxyphenyl)-2-propanol
Sun et al. [16]	Curcumin (200 mg/kg or 50 mg/kg for 3 months) Administration to APP/PS1 double transgenic mice	Gut microbiota Demethylation, demethoxylation, reduction, hydroxylation	Dihydrocurcumin, hexahydrocurcumin, demethoxylated-curcumin, demethoxylated-hexahydrocurcumin
Peron et al. [17]	*Curcuma longa* in dray extract (100 mg curcuminoids) for 28 days on healthy volunteers	Gut microbiota healthy volunteers	1,7-bis(4-hydroxy-3-methoxyphenyl) hepta-3,5 diolo, 5-hydroxyi-1,7-bis (4-hydroxy-3 methoxyphenyl) hepata-3-one, 5-hydroxy-1,7-bis (4-hydroxyphenyl) hepata-3-one
Hassaninasab et al. [18]	Synthetic medium with 0.5% curcumin Addition to fresh human feces	*Escherichia coli* Reduction by NADPH-depend curcumin/dihydroxycurcumi reductase enzyme	Tetrahydocurcumin
Jazayeri et al. [20]	Basal TPY media supplemented with various concentrations of curcumin (2.5 mg, 5 mg, 7.5 mg, 20 mg/10 mL)	*Bifidobacterium longum BB536*, *Bifidobacterium pseud cantelanum G4*, *Enterococcus faecali JCM 5803s*, *Escherichia coli K-12*, *Lactobacillus acidophilus*, *Lactobacillus casei*	50% curcumin metabolized in polyphenol derivates
Herath et al. [21]	Curcumin	*Pica anomala* ATCC 20170	5-hydroxy-7-(4-hydroxy-3-methoxyphenyl)-1-(4-hydroxyphenyl) hepta-3-one
An et al. [22]	Food additive curcumin in mice	*Bacillus megaterium DCMB002* Hydroxylation, demethylation, demethoxylation end other reactions	1,7-bis(4-hydroxyphenyl)hepta-3,5 diolo, 5-hydroxy-1,7-bis(4-hydroxyphenyl)hepta-3-one, 5-hydroxy-1,7-bis(4-hydroxyphenyl)heptan-3-one, 5-hydroxy-7-(4-hydroxyphenyl)-1—(4-hydroxyphenyl hepta-3-one) Curcumin, Tetrahydrocurcumin, Dihydrocurcumin

The GM has a profound impact on the curcumin biotransformation into metabolites with specific biological activity. The different microbial compositions between individuals can explain the biotransformation of polyphenols differently. Furthermore, curcumin fermentation by the GM also defines how the pharmacological effect of the molecule can be linked to the presence of active metabolites of fermentative origin rather than the presence of blood metabolites [2]. This characteristic explains the inconsistency between the low blood availability of curcumin and high therapeutic efficacy, as well as the safety of the use of the substance as a therapeutic agent.

## 4. Effects of Curcumin on Gut Microbiota

Its poor bioavailability, as well as its presence and its derivatives in the gastrointestinal tract, have led to the hypothesis that the therapeutic capacity of curcumin was due to the regulatory effect of the polyphenol itself on GM. Numerous recent studies have reported the therapeutic effect of curcumin on the ability to restore eubiosis in the treated organism. They suggest that curcumin acts as a promoter of the growth, proliferation, and survival of beneficial strains or, instead, inhibits the growth of pathogenic strains belonging to the GM of the host to which it is administrated [24]. Once it reaches the intestine, curcumin modifies the existing microbiota. There are many mechanisms proposed to explain the effect of the spice on GM [25]. Among these, the most intuitive one sees curcumin facilitating the consumption of the same nutrients by bacteria [24,25,26]. A further mechanism of GM could be linked to the ability to increase the abundance of *Lactobacilli* strains, which, by producing lactic acid, can reduce the proliferation of pathogens and, with them, the formation of lipopolysaccharides (LPS). In the case of the same strains of *Lactobacilli*, such as *L. johnsonii La1* and *L. plantarum ACA-DC 287*, there is the simultaneous production of lactic acid and bacteriocin, capable of further selecting the abundance of the same pathogenic bacterial strains [21,27]. In fact, as a final effect, curcumin administration determines a shift of the GM in favor of beneficial bacterial strains, such as *Bifidobacteri*, *Lactobacilli*, and butyrate-producing bacteria, which simultaneously reduce the abundance of pathogens, such as *Prevotellaceae*, *Coriobacteriales*, *Enterobacteria*, and *Rikenellaceae*, whose flowering is associated with many metabolic diseases [2,13,24].

The majority of the studies were performed on animal models. Table 2 shows a summary of curcumin’s effect on GM composition.

### Preclinical and Clinical Studies

Zhai et al. [28] were among the first researchers to notice how, in ducks, the curcumin administration (400 mg/kg) was able both to reduce the hepatic oxidative damage induced by ochratoxin and, through the sequencing of 16S rRNA gene to induce a greater variety, in terms of bacterial species, of the GM, including butyric acid-producing bacteria. Similarly, using curcumin nanobubbles (3.0–14.4 g/kg/day) in ICR mice for 6 weeks and sequencing the 16S rRNA gene, Chen et al. [9] demonstrated an increase in the *Firmicutes/Bacteroidetes* ratio and a decrease in pathogenic strains, such as *Bacteroidetes*, *Clostridiales*, *Allobaculum*. In another study [29], supplementation with curcumin (200 mg/kg/day for 6 weeks) in rats subjected to a high-fat diet (HFD) led to the reduction of at least 36 bacterial strains potentially related to microbiota pathologies, thus proving also protective against non-alcoholic fatty liver disease (NAFLAD). It was noted that the growth of the bacteria was accompanied by the production of short-chain fatty acids (SFCA) [29]. Products resulting from microbial fermentation, such as butyric acid, were likely responsible for the immunomodulatory activity performed by curcumin, helping to alleviate low-grade inflammation, insulin resistance, obesity, and NAFLD [2,6]. In fact, curcumin (99% pure) and curcuminoids (demethoxycurcumin, bisdemethoxycurcumin) in colonic epithelial cells (T-84) caused a disruption of interferon-gamma (INF-γ) signaling within colonocytes, as reported in Midura-Kiela et al.’s study [2,6,30]. 

Shen et al. [31] demonstrated how 15 days of curcumin treatment (100 mg/kg/die) influenced the abundance of families, such as *Prevotellaceae*, *Bacteriodaceae*, and *Rinkellaceae* of the microbiota of C57BL/6 rats [24], that are the strains widely associated with the onset of Alzheimer’s, as demonstrated on APP/PS1 mice [2,6,23,32,33]. Likewise, curcumin administration (100 mg/kg/die for 12 weeks) corrected the dysbiosis caused by estrogen reduction in ovariectomized mice [3,6,30,31,34]. The effect has been linked to curcumin’s ability to increase the number of *Serratia* species, *Shewanella*, *Pseudomonas*, *Papillibacter,* and *Exiguobacterium*, to decrease the abundance of *Anaerotruncus* and *H. pilory*, as well as to reduce colon cancer in mice when administered in doses of 8-162 mg/kg/day (increasing *Lactobacillales* and reducing *Corionobacteriales* and *Ruminococcus*) [2,6,23,33,35]. In animals affected by *T. gondii*, treatment with polyphenols, such as resveratrol or curcumin, or drugs, like simvastatin, also resulted in the reduction of pro-inflammatory *Enterobacteri* and *Enterococci*, and growth of *Lactobacilli* and *Bifidobacteri*, with anti-inflammatory activity [6,33,36].

Lopresti et al.’s study was conducted on adults with self-reported digestive complaints with curcumin (curcugen 500 mg), reporting a reduction in *Bacteroidetes*, and an increase in *Firmicutes*, with diversification in *Bifidobacterium*, *Faecalisbacteria*, *Clostridium*, *Enterobacteriaceae* species [37]. Zhang et al. demonstrated an increase in GM diversity and richness with the use of curcumin (Sigma M52509) in c57Bl/6 mice. The use of polyphenol also caused the flowering of *Lactobacilli*, *Bifidobacteri*, butyrate-producing bacteria, and *Muribacullaceae* and a reduction in pathogen strains such as *Enterobacteri*, *Prevotellaceae*, and *Rikenellaceae* [2,6,33,37,38]. Finally, treatment with curcumin (98% pure, 300 mg/kg/die) also resulted in the correction of dysbiosis induced by plasma LPS, favoring both an increase in *Enterococcus* and *Butyricicoccus* and a reduction in *Ruminococcaceae* and *Alisteps* [39]. Dietary supplementation allowed a decrease in the effects of LPS produced by pathogens and, therefore, a reduction in damage to intestinal tight junctions and in the acute inflammatory response induced by the production of pro-inflammatory cytokines, such as interleukin (IL)-1β, tumor necrosis factor-α (TNF-α) and farnesoid X receptor (FXR) inhibition [39]. All these actions, which are later explained in more detail, correspond to the reorganization of the GM carried out by curcumin.

Among the clinical studies, Peterson et al. [40] considered the effect of curcumin in modifying the diversity of GM. The authors treated healthy subjects with turmeric or curcumin (6000 mg/day plus piperin) for 8 weeks, at the end of which turmeric- and curcumin-treated groups showed an average increase of 7 and 69% in detected species in the GM, respectively, compared to reduction by 15% in the placebo group. An increase in *Clostridium*, *Bacteroides*, *Citrobacter*, *Chronobacter*, *Enterobacter*, *Enterococcus*, *Klebsiella*, *Parabacteroides*, and *Pseudomonas*, and a reduction of several species of *Blautia* and most of *Ruminococcus* were found.

**Table 2:** Summary of curcumin effects on gut microbiota.

Reference	Curcumin Dosage	Gut Microbiota
Chen et al. [9]	Nano-bubble curcumin extract (3.0 g/kg/day and 15.4 g/kg/day) for 6 weeks in ICR mice	↑ *Firmicutes*, *Lactobacillus*, *Lactobacillaceae* ↓ *Bacteroidetes*, *Clostridiales* ↓ *Allobaculum*
Zhai et al. [28]	Curcumin (400 mg/kg/die) in ducks	↑ butyric acid-producing bacteria ↑ GM α diversity ↑ GM richness
Feng et al. [29]	Curcumin (200 mg/kg/die) for 6 weeks in rats undergoing HFD	↑ producing-SFCA bacteria (*Blautia*, *Allobacum*) ↓ *Spirocaertae*, *Tenericutes*, *Elusimicrobia*
Midura-Kiela et al. [30]	Curcumin (99% pure) and curcuminoids (demethoxycurcumin, bisdemethoxycurcumin) in colonic ephitelial cells (T-84)	↑ butyric acid-producing bacteria
Shen et al. [31]	Curcumin (100 mg/kg/die) for 15 days in C57BL/6 rats	↓ *Prevotellaceae*, *Prevotella* ↑ *Bacteriaceae*, *Rinkellaceae*, *Alisteps*, *Bacteroides*
Zhang et al. [34]	Curcumin (100 mg/kg/die) for 12 weeks in ovariectomized mice	↑ *Serratia*, *Shewanella* ↑ *Pseudomonas*, *Papillibacter*, *Exiguobacter* ↓ *Anaerotruncus*, *Helicobacter pylori*
McFadden et al. [35]	Curcumin supplement diet (curcumin 98.01% pure with curcumindoids demethoxycurcumin, bisdemethoxycurcumin) in for 30 weeks in 129/SVEV mice and germ-free Il 10^−^/^−^ mice	↑ *Lactobacillus* ↓ *Corionobacteriales*, *Ruminococcus*
Bereswill et al. [36]	Curcumin (20%) in murine model C57BL/10, affected by *T. gondii*	↓ *Enterobacter*, *Enterococii* ↑ *Lactobacillus*, *Bifidobacteria*
Lopresti et al. [37]	Curcugen (curcumin extract 500 mg) in adults with self-reported digestive complaints	↓ *Bacteroides* ↑ *Firmicutes* Variation of *Bifidobacterium*, *Faecalibacterium*, *Clostridium*, *Enterobacteriaceae*
Zhang et al. [38]	Curcumin (Sigma M5250) in C57BL/6 mice	↑ *Lactobacilli*, *Bifidobacteria* ↑ butyrate-producing bacteria ↑ *Muribacullaceae* ↓ *Enterobacteria*, *Prevotellacceae*, *Rikenellaceae*
Ruan et al. [39]	Curcumin (98% pure, 300 mg/kg/die) in 1-olf-day chickens	↓ *Ruminococcaceae*, *Alisteps* ↑ *Enterococcus*, *Butyricicoccus*
Peterson et al. [40]	Curcumin (6000 mg/day plus piperine) for 8 weeks in healthy subjects	↓ *Blautia* sp. ↑ *Clostridium*, *Bacteroides*, *Citrobacter*, ↑ *Cronobacter*, *Enterobacter*, *Enterococcus*, ↑ *Kleibsiella*, *Parabacteroides*, *Pseudomonas*

HFD—high-fat diet; SFCA—short-chain fatty acids; GM—gut microbiota.

## 5. Dysbiosis and Glycemic Homeostasis

Several studies associate dysbiotic GM with the pathophysiology of many chronic diseases, such as T2DM, MS, and obesity, as well as glucose and lipid metabolism [41]. Similarly, numerous studies have been published on the effectiveness of curcumin in regulating blood sugar [42]. These observations, together with the large curcumin and its metabolites presence in the intestine after oral intake, support the hypothesis that the remodeling that curcumin operates on the GM could be among the factors determining the therapeutic activity carried out by the polyphenol in the host. Moreover, GM acts through the control of metabolic pathways shared with the host, interfering with its health. GM acts mainly by producing products and metabolites generated by the fermentation of compounds received from the diet, whose components include polyphenols and, therefore, curcumin. These molecules, produced by GM through its enzymatic fermentation, are capable of inducing genetic and epigenetic modifications, changes in neuroendocrine secretion, and the permeability of the intestinal membrane. Among these substances, there are plasma LPS, bile acids, metabolites of choline, tryptophan, the products deriving from fermentation of carbohydrates and proteins, such as SFCA, branched-chain amino acids, aromatic amino acids and many others, including trimethylamin N-oxide [43]. Each of these substances activates specific pathways in the host that are capable of determining pro- or anti-inflammatory actions, which can induce or protect against the risk of metabolic pathologies, including hyperglycemia, MS, and T2DM. Therefore, dysbiosis is associated with and may be the cause and aggravation of many human diseases, as confirmed by the most recent studies [2]. This places GM and its possible modification as a target for the treatment and prevention of metabolic pathologies. Therefore, it is important to identify both the eubiotic GM and the phenotype associated with metabolic diseases, together with these factors capable of modifying its asset, such as curcumin.

Regarding the hyperglycemic phenotype, a review of 42 clinical observational studies identified the same microbial genera belonging to the GM and negatively associated with T2DM. These genera include *Bifidobacterium*, *Bacteroides*, *Faecalibacterium*, *Akkermansia*, and *Roseburia*. Others, however, such as *Ruminococcus*, *Fusobacterium*, and *Blautia,* become part of the GM in the diabetic and hyperglycemic subjects. *Lactobacillus* shows its own strain specificity and appears to carry out protective actions in synergy with *Bifidobacterium*. For example, while in the patient with T2DM, the strains of *L. acidophilus*, *L. gausseri*, and *L. salivarius* are increased, *L. amylovorus* seems reduced [41].

In T2DM patients, there is an evident lower abundance of butyrate-producing bacteria, such as the class of *Clostridia*, the *Faecalisbacterium* genus, and the *Firmicutes* phylum [44]. Each of these single genera, species, and strains belonging to the host’s GM will be able to ferment the food products received with the diet, including polyphenols and thus, curcumin. The resulting molecules represent the meeting points between the GM and the host and influence each other. Indeed, these molecules can promote or inhibit metabolic pathways shared between the GM and the organism. Then, the GM produces specific metabolites that can enter the host’s metabolic pathways. These include the immunomodulation pathways, maintenance of intestinal permeability, and the homeostasis of glucose and lipids in major organs.

Regarding the immune pathways, some GM species favor the production of metabolites that facilitate the release of anti-inflammatory mediators, while others act by inhibiting their release and vice versa. For example, the same genera, such as *Bacteroides*, *Akkermansia*, *L. plantarum*, and *L. casei*, induce the production of IL-10, a cytokine with anti-inflammatory action. IL-10 is known to protect skeletal muscle from insulin resistance [38,41,45]. *R. intestinalis* increases the release of IL-22, an anti-inflammatory cytokine capable of modulating insulin resistance, the differentiation of regulatory T-cells, and Transforming Growth Factors (TGF-β) production [46,47]. Likewise, *B. thetaiotaomicron* induces T-reg cell gene expression. TGF-β plays an important role in the function, proliferation, apoptosis, and differentiation of pancreatic β-cells, the dysfunction of which leads to diabetes. In this way, depending on the predominant strains, GM has a direct action in the immunomodulation, inflammation, and glycemic and lipid host metabolism [46]. In other cases, however, the same microbial genera inhibit the release of pro-inflammatory cytokines. An example is given by *L. plantarum*, *L. paracasei*, and *L. casei*, which decrease IL-1β, monocyte chemoattractant protein-1, cell adhesion molecule, IL-8, Cluster of Differentiation 36 (CD36) and C-reactive protein [48]. *L. paracasei* and *B. fraglis* also inhibit IL-6, while some strains of *Lactobacillus*, *Bacteroides*, and *Akkermansia* limit TNF-α, as well as *L. paracasei*, *F. prausnitzii*, *Roseburia*, and *Faecalibacterium* act by inhibiting Nuclear factor-kappa-light-chain-enhancer of an activated B cell (NF-kB) [41,49,50]. All these molecules are involved in the control of insulin resistance. For example, IL-1β influences insulin secretion; IL-6 stimulates glyconeogenesis and gluconeogenesis; IL-8 and TNF-α impair insulin receptor-mediated signaling.

The GM can also interact with the host by controlling the permeability of the intestinal barrier. In fact, a functionally active intestinal membrane allows for low-grade inflammation possibly caused by LPS. LPS, deriving and produced by pathobionts presented and hosted in the GM itself, can cause endotoxemia. Some in vivo studies investigated how many and which strains regulate the expression of genes involved in the integrity of the intestinal membrane [41,45,51,52]. 

The GM can also directly influence glucose metabolism by modulating the expression of glucose transporters. For example, the *B. lactis* strain modulates the expression of hepatic gluconeogenesis, insulin-stimulated glucose adsorption, and also the translocation of Glucose Transporters (GLUT)-4. The action on GLUT-4 is also operated by *L. gasseri* BNR-17, which increases its expression in the muscle with a hypoglycemic effect [53]. SFCAs, produced by commensal butyric acid-producing bacteria, act through the G-protein receptors (GPR)41 and GPR43 receptors, regulating the expression of glucose-dependent co-transporter-1 (SLG-1) and GLUT-2, thus influencing postprandial glucose adsorption [54]. 

Furthermore, some specific species control the genes of many glycemic homeostasis key enzymes. *L. casei* improves insulin resistance using phosphatidylinositol-3-kinase (PI3K) pathway, the 5′-AMP-Actived Protein Kinase Catalytic Submit-2 (AMPK2), Protein Kinase B (Akt2 o RAC-beta serine/threonine-protein chinase), and controls the synthesis of hepatic glycogen [41]. Likewise, the same strain reduces hyperglycemia in Caco-2 cells through a biliary pathway involving the regulation of many genes, the insulin-degrading enzyme, and the growth factor-like binding protein insulin (IGFBP-3) in white adipose tissue [55]. *Lactobacilli* and *Akkermansia* reduce postprandial hyperglycemia by inhibiting α-glucosidase, which degrades complex carbohydrates [56].

The GM can also modulate the secretion of enterohormones. For example, the butyrate-producing bacteria, *Bifidobacterium* and *Lactobacillus* promote the release of Glucagon Like Peptide (GLP-1), GLP-2, and Peptide YY (PYY) from enterochromaffin L cells. *Bifidobacterium*, in fact, exploits the GPCR41 and GPCR43, while *Lactobacillus* activates the G-protein coupled bile-acid receptor (BA/TGR5) pathway [41,57]. The SFCA produced by these strains also activates the GPR41 and GPR43 receptors, which are agonists, promoting further secretion of GLP-1, resulting in insulin release and improvement of glucose homeostasis.

**Figure 3:**
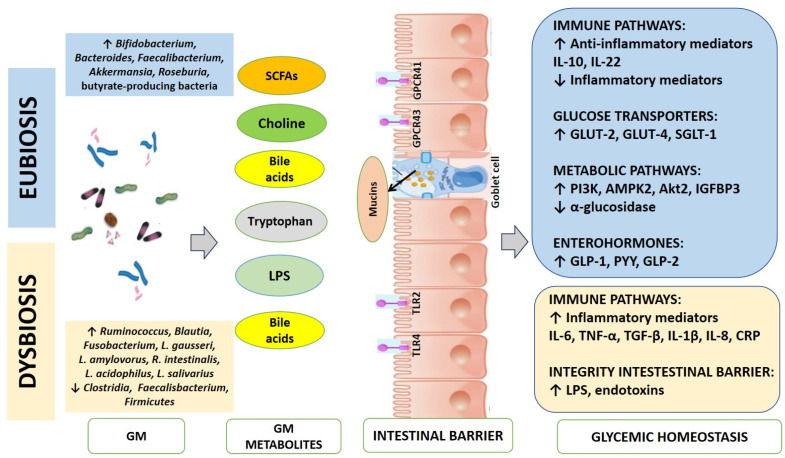
Microbiota influence on glycemic homeostasis. Depending on its bacterial composition, the gut microbiota (GM) produces various molecules, such as bile acids, choline, and tryptophan, capable of inducing changes in intestinal membrane permeability and activating several pathways that regulate glycemic homeostasis. In eubiosis, GM regulates mucins production by goblet cells to protect the intestine and increases the expression of SGLT-1, GLUT-2, and GLUT-4 transporters on epithelial cells, which, in turn, regulates the balance between glucose uptake. Moreover, it promotes, through the activation of GPR41 and GPR43 receptors, the release of GLP-1. SCFAs promote insulin secretion through the vagus nerve. The activation of all these pathways determines a reduction in hyperglycemia. In dysbiosis, GM activates TLR2 and induces the release of TNF-α and IL-6 by dendritic cells. GM releases LPS to destroy the intestinal barrier and stimulates the activation of TLR4, increasing local inflammation. Increased intestinal permeability and elevated levels of inflammatory mediators are characteristics of type 2 diabetes. LPS—lipopolysaccharides; SCFAs—short chain fatty acids; GPCR—G-protein coupled receptor 43.; TLR—Toll-like receptor; IL—interleukin; TGF-β—transforming growth factor-β; CRP—C-reactive protein; TNF-α—tumor necrosis factor-alpha; SGLT-1—sodium-glucose cotransporter 1; GLUT—glucose transporter; PI3K—phosphoinositide 3-kinase; AMPK2—AMP-activated protein kinase; Akt2—Serine/Threonine Kinase-2; IGFBP3—insulin-like growth factor-binding protein 3; GLP—glucagon-like protein; PYY—peptide YY.

Figure 3 shows the GM influence on glycemic homeostasis both in eubiosis and dysbiosis.

## 6. Curcumin Effect on Glycemic Homeostasis

The hypoglycemic effects of curcumin are now known, so much so that it is considered a potential natural antidiabetic agent [42]. However, the mechanism through which curcumin exerts its hypoglycemic action has not yet been well identified and defined. The action of polyphenol consists of improving the function and survival of pancreatic β cells, increasing insulin secretion, improving glucose adsorption in adipocytes and skeletal muscle cells, and inhibiting gluconeogenesis and inflammation in liver and adipose cells [1]. Furthermore, curcumin, thanks to its activity on the GM, is able to control the host’s glucose homeostasis through mechanisms that include an improvement in the barrier function, the secretion of enterohormones, anti-inflammatory cytokines, and the reduction of many inflammatory molecules, involved in the pathogenesis of insulin resistance. Curcumin, in fact, can modify the richness and diversity of GM with notable increases. Administered orally to mice and other animal models, curcumin increases the number of beneficial bacteria (*Lactobacilli*, *Bifidobacteria*, and butyrate-producing bacteria) and simultaneously reduces pathogenic bacteria (*Enterobacteria*, *Prevotellaceae*, and *Rikenellaceae*). In particular, its administration increases the abundance of the *Muribaculaceae* family, bacteria producing succinic acid, acetic acid, and propionic acid, the reduction of which is linked to the onset of inflammatory intestinal pathologies and type I diabetes [38].

**Figure 4:**
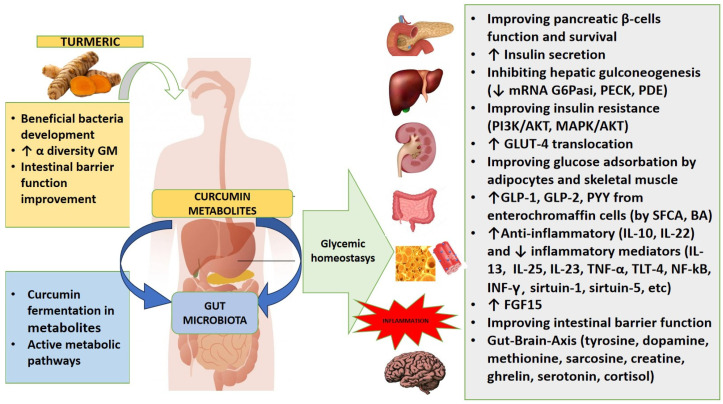
Summary of the effect of curcumin on glycemic homeostasis.

Numerous in vitro and in vivo studies conducted in recent years have identified various possible mechanisms of action carried out by curcumin in the regulation of glucose homeostasis and insulin secretion and signaling [1]. However, the number of studies conducted on humans remains quite small. Figure 4 shows a summary of the effect of curcumin on glycemic homeostasis.

The first mechanism of action exerted by curcumin on glycemic control was identified by Rouse et al. [58]. Curcumin induced a down-regulation, according to a dose-dependent manner, of the mRNA expression of at least 11 phosphodiesterases (PDEs) isoenzymes, including PDE3B, PDE8A and PDE10A. These naturally suggested how the polyphenol can act, influencing the regulation of the PDE/cAMP pathway [1,58]. Studies in treated hepatocytes, curcumin reduced gluconeogenesis and glyconeogenesis through a reduction in the activity of both hepatic glucose-6 phosphatase (G6Pase) and phosphoenolipyruvate carboxykinase [1,59]. Many of the observations made by in vitro studies were reconfirmed in vivo experiments. Curcumin has also been shown to reduce G6Pase in studies conducted on albino Wistar rats or C57BL/6 or C57/BL6J mice with diabetes streptozotocin- or HFD-induced diabetes [60,61,62]. Curcumin also reduced Mitogen-Activated Protein Kinase (MAPK) mediated angiotensin-converting enzyme expression and Ras Sarcoma virus activation. This involves the establishment of normoglycemia and the maintenance of pancreatic GLUT-2, a significant reduction in glycated hemoglobin (HbA1c), an improvement in insulin resistance and lipid profile, the reduction of leptin and the increase of adiponectin, and inhibition of the NLP3 inflammasome, with a global significant decrease in low-grade inflammation [1]. In mice fed an HFD, curcumin mitigated fasting blood sugar, glucose intolerance, and insulin resistance [63]. It also inhibited the hepatic gluconeogenesis. The study highlighted how polyphenol reduced the mRNA expression levels of phosphoenol pyruvate kinase (PCK1) and G6Pase catalytic submit 1, which, therefore, encode two key enzymes involved in hepatic gluconeogenesis. The expression of genesis involved in hepatic de novo lipogenesis is also reduced [64]. 

Furthermore, as highlighted by in vitro and in vivo studies on murine models, curcumin and, even more so, its derivatives, such as THC, demethoxycurcumin, octahydrocurcumin, and bisdemethoxycurcumin can access the phosphotidilinositol 3-kinase/protein kinase B (PI3K/Akt o PI3K/PKB) pathway, which together MAPK/Akt, represent the main signal transduction system sensitive to oxidative stress and responsible for cell grown and death. It also acts both the AMP-activated protein kinase (AMPK) pathway, which regulates energy metabolism and cellular homeostasis, and the nuclear factor E2-related factor (Akt/Nrf2) pathway, capable of up-regulating the antioxidant mechanism involved in the metabolism of reactive oxygen species [3,6,65,66,67,68]. The same mechanism is also carried out by the GM, which thus becomes capable of interfering with the regulation of blood sugar. For example, the *L. casei* strain, whose abundance is favored by curcumin, improves insulin resistance precisely through the PI3K, AMPK2, Akt2, and hepatic glycogen synthesis pathways [41]. *L. casei* also reduces hyperglycemia through a biliary pathway involving the regulation of many genes, such as ClC1-7, Glycine Receptor Antibodies-α1, Solute Carrier Family (SLC) 26 member (A) 3, SLC26A6, gamma-aminobutyric acid type A receptor subunit alpha 1, Bestrophin-3, and Cystic Fibrosis Transmembrane Regulator. It also reduces insulin-degrading enzymes in Caco-2 cells and IGFBP-3 in white adipose tissue [41]. In in vitro experiments conducted on skeletal muscle cells, hepatocytes, and adipocytes, curcumin treatment not only improved the phosphorylation of Akt but determined an increased GLUT-4 translocation, and a reduced pro-inflammatory cytokines production [1]. There are therefore other mechanisms through which curcumin can interfere with glucose homeostasis. However, curcumin is able both to inhibit the reabsorption of glucose in fibroblasts by binding to GLUT-1 transporters in an immediate, non-additive, and reversible way and to regulate, in hypoxic adipocytes, the expression of GLUT-1 (down-regulated) and GLUT-4 (up-regulated) [64,69]. Given the controversial effects on glucose transporters, the effect has been linked to dose and exposure time; in this sense, chronic treatment could induce an up-regulation of the expression of GLUT proteins by compensation [1]. The administration of 80 mg/kg/day (for 8 weeks) of curcumin to obese albino male Wistar rats suffering from T2DM resulted in an improvement in glycemic parameters, establishing lower insulin resistance, hypolipidemia, and reduction of malondialdehyde levels in the liver and pancreas [70]. The hypoglycemic activity has been related to an increase in the GLUT-4 gene [7]. 

GM itself can directly influence glucose metabolism by favoring the expression and translocation of GLUT-4. For example, *B. lactis*, increased by curcumin, promotes glycogen synthesis and decreases the expression of hepatic gluconeogenesis genes, improving glucose adsorption stimulated by insulin and the translocation of GLUT-4. *L. gasseri* BNR-17 also increased the expression of GLUT-4 in the muscle with an evident hypoglycemic effect [41].

Curcumin also influences blood sugar levels by increasing the secretion of GLP-1. The mechanism by which curcumin facilitates the release of incretin hormone could be linked to an inhibition of the activity of dipeptylpeptidase-4, i.e., a surface glycoprotein that degrades GLP-1, as demonstrated on Caco-2 cells [71]. Differently, curcumin seems to directly stimulate the secretion of GLP-1 by activating the Ca^2+^/calmodulin-dependent kinase II pathway (independently of the cAMP/PKA pathway) [72]. Both these mechanisms can however be explained and correlated to microbial activity [72,73,74]. Furthermore, the involvement of the GM would also explain the need to administer curcumin for a sufficiently long period to thus allow the time required for the modification and proliferation of the GM [5].

Confirming what had also been observed in in vitro studies, curcumin appears to act in animal studies by promoting the release of the incretin hormone GLP-1. The administration of THC for 8 weeks in db/db mice reduced glycemia, increased insulin secretion, and the expression of GLP-1 in the pancreas [5]. Furthermore, the analysis of the sequencing of the 16s rDNA of the intestinal microbiota shows the ability of THC to restore the dysbiosis associated with diabetes, varying the relative abundance of *Proteobacteria* and *Actinobacteria*, negatively associated with the enteriohormone release, and the ratio between *Firmicutes* and *Bacteroides*, which are positively associated with GLP-1 secretion [5]. This, therefore, raises the hypothesis that one of the mechanisms by which curcumin and its analogs operate a hypoglycemic effect is linked to the regulatory effect on the GM and the growth caused by them by the presence of SFCA-producing bacteria. SFCA, through their GPR43 and GPR4 receptors, can influence the secretion of GLP-1 and consequently increase insulin release and improve glycemic homeostasis [1,2,7,73]. Even in diabetic rats, THC is able to modulate blood glucose levels, GLP-1 expression, β-cells of pancreatic islet protection, and insulin secretion. Even in this case, the hypoglycemic function was related to the restoration of intestinal dysbiosis. In fact, the metabolite restores the relative abundance of *Actinobacteria*, *Proteobacteria*, and the *Firmicutes/Bacteroides* ratio [5].

The oral administration of highly dispersible curcumin in the form of nanoparticles determined a secretion of GLP-1 by GLUTag cells. [8] The polyphenol’s secretion of the incretin hormone was mainly due to the stimulation of the GPCR40 and GPCR120 receptors, with a consequent hypoglycemic effect linked to improved insulin secretion. Curcumin acted as a natural agonist of TGR5 and FXR by improving the release of GLP-1 by expansion of intestinal L cells. Furthermore, if administered to ob/ob mice for 8 weeks, curcumin improves obesity and glucose tolerance by increasing energy expenditure. Therefore, through the mycobiota-intestinal-TGR5/FXR axis, polyphenol becomes a good therapeutic agent for metabolic disorders [73,74]. In fact, butyrate-producing bacteria, *Bifidobacterium* and *Lactobacillus*, favor the release of GLP-1, PYY, and GLP-2 from enterochromaffin L cells. The former acts through GPCR41 and GPCR43, whereas the latter acts through the production of bile salt hydrolases, which produce secondary bile acids capable of activating TGR5 receptors [41]. Thus, a further metabolic pathway with which curcumin can interact, through GM, on carbohydrate and lipid metabolism appears to be the bile acids/FXR pathway. 

In addition to the ability to produce SFCA, curcumin, by acting on *Bacteroides* equipped with bile salt hydrolase, can influence the metabolism of bile acids [63]. The administration of curcumin to 1-day-old chickens allowed the restoration of dysbiosis caused by exposure to LPS, increasing the presence of *Butyricicoccus* and *Enterococcus* and reducing both *Ruminococcaceae* and *Alisteps* [39]. This allowed an increase in the concentrations of butyric acid, 3-hydroxybutyric acid, malic acid, and succinic acid, with a simultaneous reduction of alanine. As a consequence, succinate increased the secretion of IL-22, IL-25, and IL-13 from group 3 innate lymphoid cells of the intestinal lamina and suppressed IL-23, linked to up-regulation of the expression of genes controlling the acid cycle, tricarboxylic acid, IL-1β, and TNF-α. The correction of dysbiosis operated by curcumin also allowed an improvement in the concentration of the primary and secondary metabolites of bile acids (chenodeoxycholic acid and lithocholic acid). Curcumin supplementation increased the expression of the ileal gene for FXR protein. Furthermore, secondary Bas can interact with mitochondria, regulating the metabolism of lipids and carbohydrates through FXR and GPRC5. Curcumin also increased the expression of ileal GPRC5A and GPRC5B and the transcription and protein expression of sirtuin 1 and sirtuin 5, which in turn are involved through the bile acids/FXR signaling pathway in maintaining carbohydrates homeostasis [39].

Another molecule that may represent an interaction between GM, host, curcumin, and hypoglycemic effect is Fibroblast Growth Factor 15 (FGF15), a metabolic hormone synthesized and secreted by ileal enterocytes. FGF15 improves insulin sensitivity in glucose and lipid metabolic disorders [13]. It inhibits two key enzymes in hepatic gluconeogenesis (G6Pase and phosphoenolpyruvate carboxykinase), causing impaired glucose tolerance in FGF15 knockout mice [63]. Overexpression in FGF15, therefore, mitigates glucose intolerance and insulin resistance in HFD-exposed mice. Furthermore, the expression of FGF15 is also regulated by the FXR, confirming the involvement of GM in the hypoglycemic activity carried out by curcumin through bile acids [50].

Curcumin also appears to act by improving the permeability of the intestinal membrane and, therefore, reducing the release of pro-inflammatory cytokines. For example, in the study of Wang et al. [75] and Faralli et al. [76], curcumin administrated to Caco-2 cells was able to counteract the secretion of IL-1β and, therefore the activation of p38 MAPK and consequent phosphorylation of tight junction proteins, thus preventing some interruptions in the intestinal barrier function [2,6,75,76]. The integrity of the intestinal barrier function is necessary to avoid phenomena such as endotoxemia, capable of causing chronic low-grade inflammation, which is the primary cause of metabolic diseases such as T2DM, MS, obesity, cardiovascular disease, and even cancer. Curcumin has also been shown to have an effect on alkaline phosphatase, the maintenance of the mucus layer on the epithelium, regulating the expression of tight junction proteins, and the production of antibacterial peptides [33,37,77,78]. Treatment with curcumin corrected dysbiosis in chicken, favoring the growth of *Enterococcus* and *Butyricicoccus* and reducing *Ruminococcaceae* and *Alisteps*. Dietary supplementation allowed the reduction of the effects of LPS, the injury of intestinal tight junctions and the acute inflammatory response induced by the production of pro-inflammatory cytokines, such as IL-1β and TNF-α [39]. In this case, the GM, modified by curcumin, plays an important function in the integrity of the intestinal barrier. For example, *Bacteroides* are able to improve the intestinal tight junction of mice, *A. muciniphila* and its protein Amuc_1100 promote the expression of occluding and tight junction protein-1, while *Faecalium* and *Roseburia* reduce intestinal permeability via serotonin transporters and PPAR-γ pathways [41,45].

Curcumin can also interfere with glucose homeostasis by controlling the synthesis or the release of pro and anti-inflammatory molecules. In animal models, such as rats subjected to HFD or mice fed a Western diet, curcumin administration not only reduced LPS endotoxemia but also decreased serum TNF-α and other pro-inflammatory mediators concentrations, as well as inhibited the activation of NK-kB and its nuclear translocation [4,5,79]. Curcumin, by promoting the growth of bacteria-producing SFCA, protected rats subjected to HFD against NAFLAD onset and reduced insulin resistance through interference with an INF-γ signaling within colonocytes, thus reducing enteric inflammation [2,6,77]. Even in diabetic patients, the co-administration of piperine and curcuminoids (500 mg plus 5 mg/day for 3 months) promoted a reduction in blood sugar, C-peptide, HbA1c, alanine aminotransferase and aspartate aminotransferase. In patients with NAFLAD, co-administration of the two spices (500 mg with 5 mg/day for 12 weeks) improved lipid profiles [1]. Moreover, the inhibition of LPS production itself interrupts the TLR4/MyD88/NF-kB signaling pathways and therefore, also the low-grade inflammation responsible for metabolic pathologies.

As a proof of concept, Gan et al. [80] observed that curcumin administration for 28 days in piglets, inhibiting both *E. coli* and LPS-producing pathobionts, reduced the expression of TLR4, alleviating the intestinal inflammation [2]. Zam et al. [24] underlined how this activity was supported by the growth of SFCA and butyrate-producing bacteria in the fecal microbiota [2,6]. Some strains of *L. paracasei*, *F. prausnitzii*, *Roseburia*, and *Faecalibacterium* limit TNF-α production and NF-kB activation [41]. The co-administration of curcumin and piperine determines, in different types of cells, the inhibition of various inflammatory signaling pathways, such as TLR4, NF-kB, IL-1β, mitochondrial pyruvate carrier-1, cyclooxygenase 2, the latter with tissue-specific activity [4]. At the same time, curcumin plus piperine administration reduces TFN-α, IL-10, IL-12, p70, IL-1β, and IL-6, with a direct action both on glycemic control and GM [1]. Moreover, in a double-blind, placebo-controlled clinical study, curcumin improved glucose adsorption, suppressing NF-kB pathways and up-regulating PPAR-γ [1].

Curcumin also seems to act through the gut–brain axis. In rats with diabetic gastroparesis, curcumin improved the expression of ghrelin, another enteric hormone capable of balancing energy homeostasis. The activity was linked to the ability of the polyphenol to modify the GM towards a good relationship between benefits and pathogens, with an abundance of *Bifidobacteria* and *Lactobacilli*, and a clear reduction of strains of *Corionabacteriales*, *Prevotellaceae*, *Enterococci*, *Enterobacteriaceae,* and *Rikenellaceae* [50]. Furthermore, curcumin increases tyrosine, dopamine, methionine, sarcosine, and creatine levels, activating and regulating the gut–brain metabolic axis, and with its caloric intake and energy homeostasis. Furthermore, through studies of people suffering from irritable bowel syndrome and different gastrointestinal pathologies, curcumin, again through the modulation of the GM, influenced the hypothalamic–pituitary–adrenal activity, reducing blood concentration of cortisol and serotonergic activity [27,38].

Even in mice fed with HFD, curcumin determined an improvement in intestinal dysbiosis, which induced thermogenesis depending on uncoupling protein 1, otherwise called thermogermin or UCP1, located in the mitochondria of brown adipose tissue [63,81].

Curcumin therefore seems to control glucose homeostasis through numerous mechanisms, of which GM, modified by it, is complicit. Although limited in number, clinical studies confirm curcumin’s pharmacological activity. However, these studies are exposed to greater uncertainty. In fact, the GM undergoes important modifications both with the diet administered and through the lifestyle followed by the host and is therefore less standardizable than the animal model. In any case, all randomized, double-blind, placebo-controlled clinical studies conducted for periods of 3, 6, and 9 months showed how treatment with curcumin was able to reduce serum levels of glucose, triglycerides, low density lipoproteins (LDL), HbA1c, leptin and increase adiponectin, with protection against T2DM [82]. A study conducted in a double-blind controlled study on overweight patients with suboptimal glycemia showed how the administration of a phytosomal preparation of curcumin (800 mg/day for 8 weeks) reduces plasma insulin, the HOMA index, triglycerides, LDL, hepatic transaminases, γ-GT, blood cortisol, waist circumference, blood pressure, and hepatic steatosis [1].

## 7. Safety and Doses for Beneficial Effects of Curcumin

US Food and Drug Administration has approved curcumin (E100) as a “compound generally recognized as safe (GRAS)”. The Joint FAO/OMS Expert Committee on Food Additives and the European Food Safety Authority have defined the Acceptable Day Intake for curcumin as 0–3 g/kg^−1^ [1,2,83]. However, a European Union herbal monograph on curcumin reports that it can cause flatulence, gastric irritation, stimulation of bile secretion, and cholangitis. The cholecystokinetic effect can be aggravated by the presence of piperine with the risk of hepatotoxicity. Some cases are reported in literature and have led the Ministry of Health to impose a warning on products containing the rhizome. To date, no adverse effects on blood sugar have emerged [1,84,85]. However, although it is considered a safe product, its use should be limited in people suffering from liver diseases, such as cirrhosis, biliary obstruction, gallstones, and people who abuse alcohol. 

Additionally, curcumin may interact with non-steroidal anti-inflammatory drugs, reserpine, and anticoagulants [12]. Other studies showed that nausea, diarrhea, elevated levels of alkaline phosphatase, and serum lactate dehydrogenase may appear in subjects treated with curcumin at doses of 0.45–3.6 g/day; likewise, Shabbir et al.’s study [7] suggested that the use of curcumin up to 8 g/day was safe, while the latest animal and clinical studies raised the safety level of the polyphenol at 12 g/day [6,7,23]. In any case, to obtain a beneficial effect on humans, at least 500 mg di curcumin/day is required, which means a daily intake of 4 g of turmeric [33]. In general, therapeutic efficacy requires prolonged treatment for at least 8–12 weeks, with a curcumin dosage between 0.08 mg/kg and 1500 mg/kg/day [5].

## 8. Conclusions and Perspectives

Polyphenols have aroused great interest in the scientific community due to their high biological activity largely linked to their transformation, by the GM, into metabolites capable of carrying out intrinsic biological effects similar to or greater than the original molecule [86]. Among polyphenols, it is important to mention curcumin, already widely known for its anti-inflammatory effects and its high effectiveness in the prevention and treatment of important metabolic conditions, such as T2DM, MS, and obesity. GM, by converting curcumin into more active metabolites through its enzymatic activity, becomes the protagonist of the pharmacokinetics and pharmacodynamics of this polyphenol [87] and, therefore, takes on a fundamental role not only in the metabolism but also in its efficacy, toxicity, and bioavailability, once administrated orally to the host [88]. Curcumin and its derivatives can then directly modulate the metabolic and physiological responses of the latter, or they can exert their activity through a prebiotic-type effect on the intestinal microbial populations [88]. 

In recent years, the link between dysbiosis and metabolic pathologies has been increasingly highlighted. For this reason, GM itself and the possibility of its reworking are increasingly becoming an important therapeutic target. Curcumin influences the GM with which it comes into contact after oral administration, and, at the same time, it is metabolized by this into compounds and derivatives capable of performing greater systemic and local action compared to curcumin alone. Furthermore, the bidirectional interaction between curcumin and GM allows us to understand the pharmacology of curcumin and better interpret the paradox between its poor bioavailability and its biological activity. The metabolism of curcumin varies from subject to subject due to the different composition of the GM between individuals, resulting in a more or less prolonged metabolization of curcumin into its more active biological derivatives. Therefore, the individual composition of the GM will cause a different biotransformation of dietary curcumin. Consequently, the beneficial effects of polyphenols are not only associated with the dose consumed but also with the type of GM population that distinguishes the individual capable of fermenting it. This combination also opens a new vision of the concept of bioavailability, not only of curcumin but of every molecule with biological activity. It is called pharmacomicrobiomics.

In conclusion, preclinical and clinical studies are needed to fully understand the mechanism between curcumin and GM. Still, the current state of knowledge suggests a potential role of curcumin as a treatment to re-establish intestinal eubiosis with an effect on glycemic control. Further clinical studies are needed to confirm the effectiveness of curcumin supplementation in the treatment of metabolic pathologies, such as T2DM and MS, also in light of an individualized approach to an analysis of differences between individuals in the GM. 

## Data Availability

No data were created or analyzed in this study. Data sharing is not applicable to this article.
